# Christ-Siemens-Touraine Syndrome: A Report of a Rare Pediatric Case

**DOI:** 10.7759/cureus.60022

**Published:** 2024-05-10

**Authors:** Massilia Bouhmidi, Hajar Boudarbala, Aziza Elouali, Abdeladim Babakhouya, Rkain Maria, Noufissa Benajiba

**Affiliations:** 1 Pediatrics, Mohammed VI University Hospital Center, Oujda, MAR; 2 Pediatric Medicine, Mohammed VI University Hospital, Faculty of Medicine and Pharmacy, Mohammed I University, Oujda, MAR; 3 Pediatrics, Faculty of Medicine and Pharmacy, Mohammed I University, Oujda, MAR; 4 Pediatrics, Faculty of Medicine and Pharmacy, Mohammed I University, Centre Hospitalier Universitaire (CHU) Mohammed VI Oujda, Oujda, MAR; 5 Pediatric Hematology, Centre Hospitalier Universitaire (CHU) Mohammed VI Oujda, Oujda, MAR

**Keywords:** anodontia, hypotrichosis, hypohidrosis, anhidrotic ectodermal dysplasia, christ-siemens-touraine syndrome

## Abstract

Anhidrotic ectodermal dysplasia (AED), or Christ-Siemens-Touraine syndrome, is an X-linked recessive dermatosis. Rare in incidence, it affects 1 in 100,000 births, mostly boys. Through this observation, we detail the clinical signs that led us to suspect the diagnosis, how this pathology was confirmed, and the therapeutic management we carried out. We present a case of a 10-month-old boy presenting with altered manifestations affecting almost all the ectodermal structures like skin, hair, nails, teeth, sebaceous glands, sweat glands, and tear glands. He also had complete anodontia and a dry mouth. A multidisciplinary treatment was given to the patient with the collaboration of various health professionals. Although Christ-Siemens-Touraine syndrome is a rare condition, it is vital to recognize it early to improve care and prognosis for these patients, while mitigating the psychological impact of the condition on both children and parents.

## Introduction

Ectodermal dysplasia is a heterogeneous group of rare orphan diseases or syndromes of genetic origin characterized by an abnormality in the development or homeostasis of at least two of the following structures, all derived from the embryonic ectodermal sheet: teeth, nails, hair follicles, and certain glands. Involvement of these derivatives alone corresponds to the group of so-called pure ectodermal dysplasias. Additional involvement of another organ (eye, ear, etc.) corresponds to syndromic ectodermal dysplasia. Hypohidrotic or anhidrotic ectodermal dysplasia (HED/AED) is the most common form. It is characterized by a reduction (hypohidrosis) or absence (anhidrosis) of sweating [1,2].

## Case presentation

**Figure 1:**
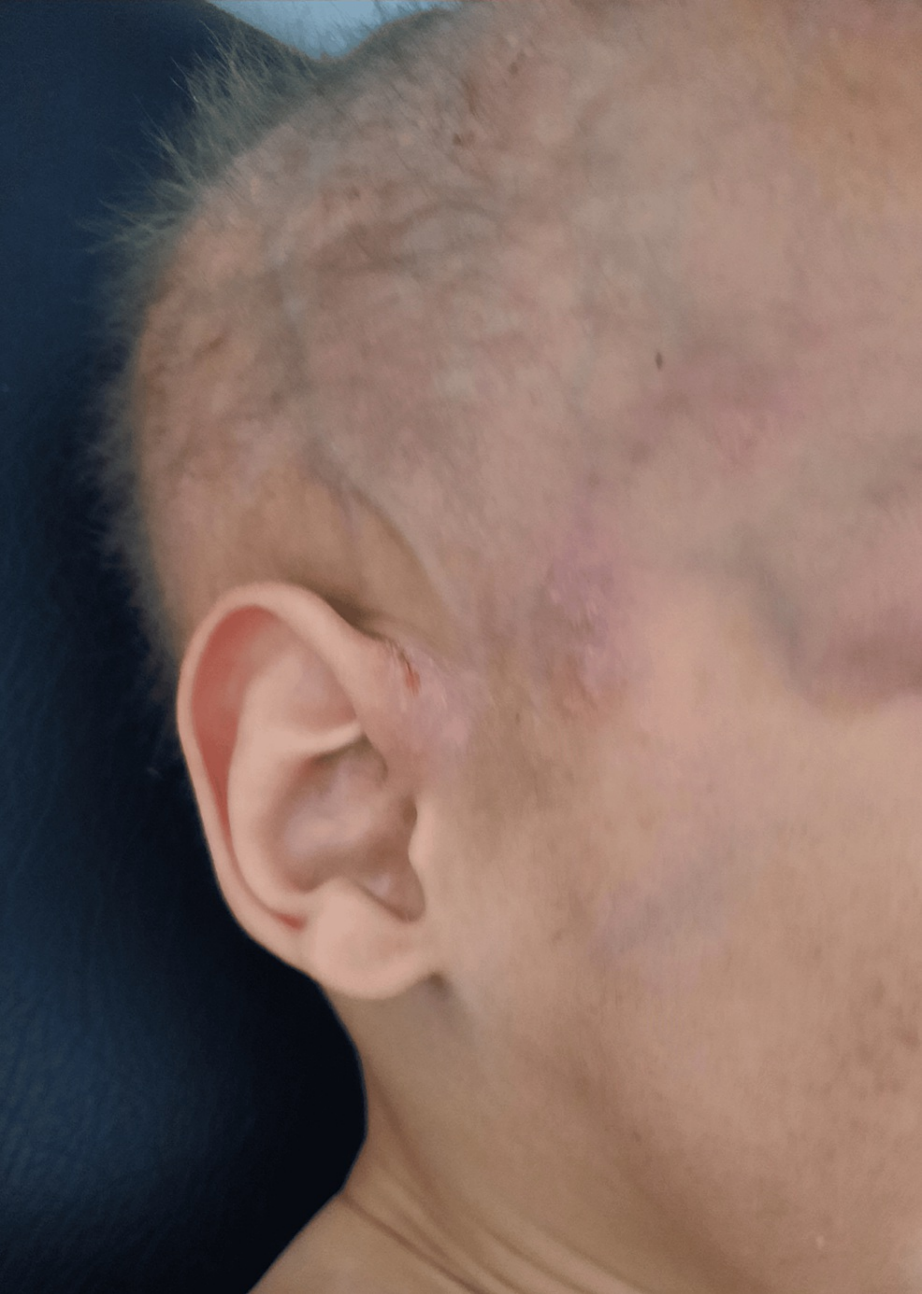
Sparse hair with a clear appearance (hypotrichosis)

**Figure 2:**
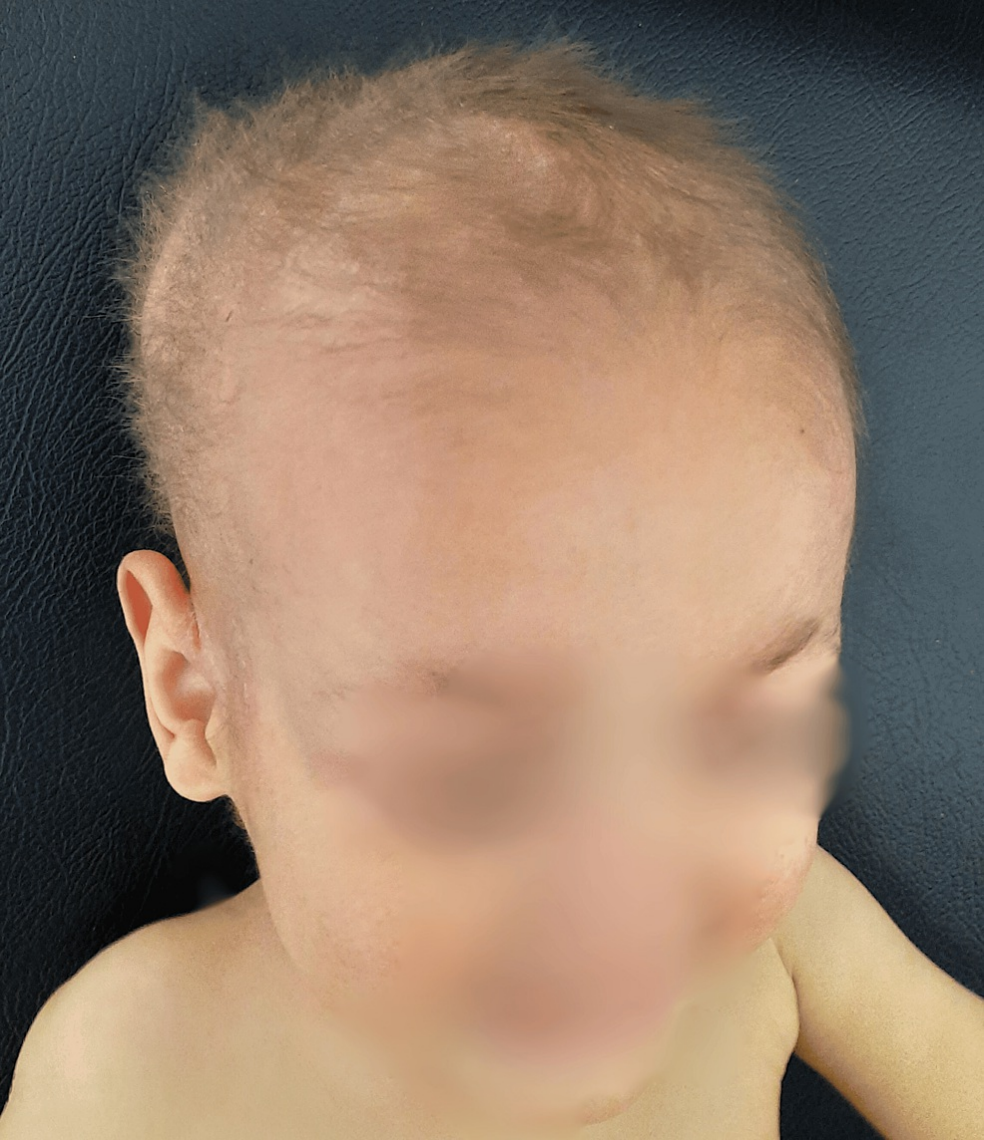
Hypotrichosis (hair, eyebrows, and eyelashes are thin and sparse)

**Figure 3:**
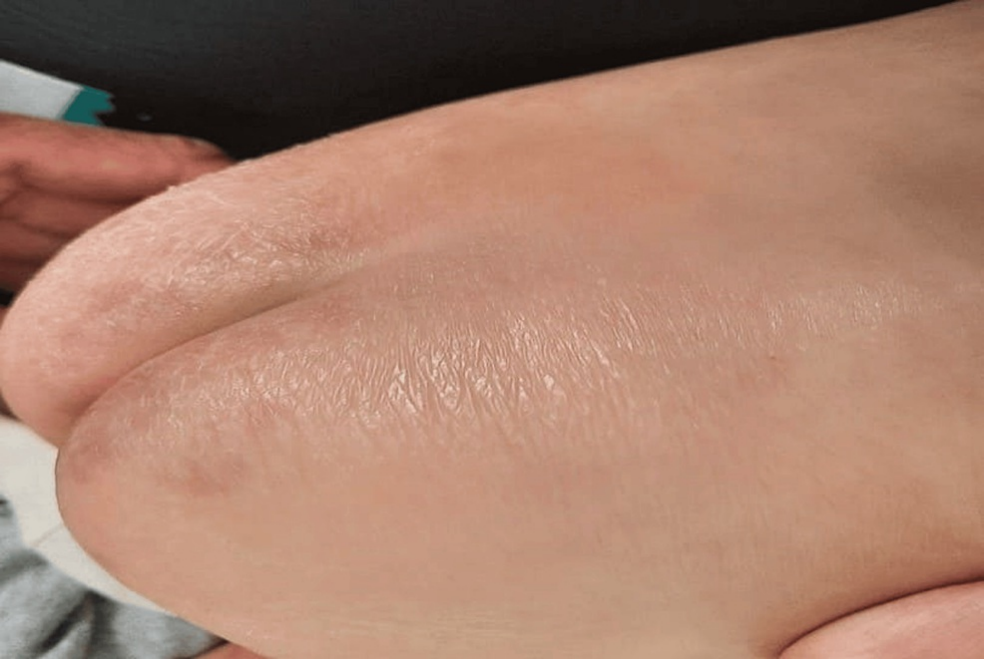
Eczematous skin with areas of hyperkeratosis in the buttocks area

A 10-month-old male infant was brought in on January 18, 2022 by his mother for recurrent fevers since the age of two months, at a rate of three episodes per month, exaggerated in summer, with a fever that had lasted for two months and was not responding to antipyretics or physical measures. His mother had noticed that since birth, he was very uncomfortable when the ambient temperature increased. This impression has been confirmed over time. Despite this hot feeling, he never sweated. The mother pointed out that his skin was periodically subject to small erythematopapular eruptions, sometimes vesicular and itchy. Scratching caused small scratches that healed quickly. The pregnancy was not monitored and the delivery was not medically assisted. He weighed 2,200 grams at birth; he was the youngest of two siblings. His older brother was doing well, with normal teeth for his age and no sweating problems. A family survey was carried out on three generations preceding our patient, and no dental anomaly has been noted to date. He was vaccinated according to the national vaccination protocol and had a delay in psychomotor development; he held his head at four months, sat up on his own at 10 months, and still did not walk. On physical examination, he was in good general condition. He weighed 7 kg (-3SD) with a height of 72 cm (-1SD). His hair was sparse and thinning, and his eyebrows and eyelashes were almost non-existent (Figures 1, 2). The skin was dry and eczematous (Figure 3), with areas of hyperkeratosis on the buttocks, hands, forearms, neck, and face, and there were numerous vesiculopustular skin lesions on the face, neck, scalp, and left forearm. There were scratching lesions on the forearm and neck. The mucous membranes and sclera were well stained, and he also had eyelid wrinkles and nasal saddle. Oral examination revealed anodontia and a dry mouth. He did not have nail abnormalities and his ears were implanted normally. This clinical aspect suggested the possibility of ectodermal dysplasia, consolidated by the history of an exaggerated sensation of heat and, paradoxically, the total absence of sweat raising suspicion of an absence or dysfunction of the sweat glands.

Therefore, the diagnosis of anhidrotic ectodermal dysplasia (AED) or Christ-Siemens Touraine syndrome was suggested. A skin biopsy was performed on the sole, histological examination revealed a total absence of sweat glands and a lack of pilosebaceous follicles. A genetic consultation clinically confirmed the diagnosis. Given the notion of recurrent fever with weight stagnation, the subtype of ectodermal dysplasia associated with an immunodeficiency was suspected, but the first-line assessment of an immunodeficiency returned without abnormalities with a lymphocyte count of 10,000/mm^3^, negative HIV serology, normal protein electrophoresis, and normal immunoglobulin levels for age.

Management was multi-disciplinary, with parental involvement to ensure avoidance of uncontrolled exposure to heat, or even to adopt physical cooling measures, such as frequent consumption of cool liquids, wetting clothes, or wearing special cooling jackets/caps. Regular follow-up was suggested to ensure early dental treatment as soon as the first teeth appear, restore dental function, and improve the appearance of the teeth. Also, orthodontic follow-up was suggested to schedule sinus lift procedures followed by insertion of denture-supporting implants.

## Discussion

Ectodermal dysplasia is a heterogeneous group of syndromes of genetic origin characterized by an abnormality in the development or homeostasis of two or more of the following structures, all derived from the embryonic ectodermal sheet: teeth, nails, hair follicles, and certain glands. HED/AED is the most common form. It is characterized by a reduction (hypohidrosis) or absence (anhidrosis) of sweating [1,2].

Anhidrotic ectodermal dysplasia or Christ-Siemens-Touraine syndrome is a rare genodermal disorder linked to the X chromosome, due to a mutation in the ectodysplasin gene (EDA1). The prevalence is 1/100,000 births [3]. It is a congenital and familial histodysplasia mainly affecting the skin and its appendages. The syndrome is characterized by hypohidrosis, hypotrichosis, and hypodontia or anodontia [4]. Hyperthermia in the first months of life is the main risk of this condition, leading to death or neuropsychological sequelae [4,5].

The typical clinical manifestation of AED is the "triad" of abnormal sweat glands, thinning hair, and abnormal tooth development. It is characterized by little or no sweating, thin, dry skin, and often eczema. The hair is frizzy and thinning. Most teeth or all teeth are congenitally missing, and the remaining teeth are conical or too small [1]. In addition, patients often have peculiar facial features, such as a prominent forehead, peripheral pigmentation, wrinkled skin around the eyes, stool-shaped nose, underdeveloped jaw, and rocker upper and lower lips [3]. Other general disorders may accompany this syndrome, such as ocular involvement, including lacrimal gland aplasia and agenesis of the lacrimal ducts [4,6]; respiratory involvement, characterized by recurrent bronchopneumonia, nasopharyngeal infections, and asthma due to hypoplasia of the seromucosal glands of the respiratory tract [4,7]; intellectual retardation, which may be associated with phonation and hearing disorders; and developmental delay in early childhood. The latter is the result of malnutrition due to dentomaxillary malformations [4,6].

The management of patients with AED must be multidisciplinary: medical and odontostomatologic. Symptomatic treatment is primarily aimed at advising parents to avoid the risk of hyperthermia in infants and young children. Symptomatic treatment of atrophic rhinitis consists of saline lavage, sometimes combined with antibiotic therapy to avoid superinfection [5,6].

From an odontostomatologic point of view, early oral and dental rehabilitation is necessary to ensure the harmonious development of the maxilla and to alleviate functional, aesthetic, and psychological disorders in children [8,9]. Prosthetic treatment should be initiated as early as possible to restore masticatory function and phonation, improve swallowing, restore adequate vertical dimension, and promote normal facial growth [10,11]. The presence of certain teeth on the arch allows the use of adjunctive prostheses, which have the advantage of preserving the alveolar bone and proprioceptive sensitivity and restoring the vertical dimension of the occlusion [6,11].

## Conclusions

Ectodermal dysplasia is an uncommon condition that results in notable aesthetic and functional challenges for those affected. Anhidrosis, a symptom of this condition, can be particularly uncomfortable, especially in warmer climates, and may result in elevated body temperatures. It is crucial to counsel parents on various physical methods to mitigate hyperthermia in children with this condition. Furthermore, the utilization of dental prostheses can effectively address feeding difficulties, which can otherwise impede proper growth in terms of height and weight.
